# Changes in gene methylation patterns in neonatal murine hearts: Implications for the regenerative potential

**DOI:** 10.1186/s12864-016-2545-1

**Published:** 2016-03-15

**Authors:** Bartosz Górnikiewicz, Anna Ronowicz, Michał Krzemiński, Paweł Sachadyn

**Affiliations:** Department of Molecular Biotechnology and Microbiology, Gdańsk University of Technology, Gdańsk, Poland; Department of Biology and Pharmaceutical Botany, Medical University of Gdańsk, Gdańsk, Poland; Department of Probability and Biomathematics, Gdańsk University of Technology, Gdańsk, Poland

**Keywords:** Heart, Methylome, Regeneration, Neonatal mouse, Transcriptome, Transcription factors, Microarray profiling

## Abstract

**Background:**

The neonatal murine heart is able to regenerate after severe injury; this capacity however, quickly diminishes and it is lost within the first week of life. DNA methylation is an epigenetic mechanism which plays a crucial role in development and gene expression regulation. Under investigation here are the changes in DNA methylation and gene expression patterns which accompany the loss of regenerative potential.

**Results:**

The MeDIP-chip (methylated DNA immunoprecipitation microarray) approach was used in order to compare global DNA methylation profiles in whole murine hearts at day 1, 7, 14 and 56 complemented with microarray transcriptome profiling. We found that the methylome transition from day 1 to day 7 is characterized by the excess of genomic regions which gain over those that lose DNA methylation. A number of these changes were retained until adulthood. The promoter genomic regions exhibiting increased DNA methylation at day 7 as compared to day 1 are significantly enriched in the genes critical for heart maturation and muscle development. Also, the promoter genomic regions showing an increase in DNA methylation at day 7 relative to day 1 are significantly enriched with a number of transcription factors binding motifs including those of Mfsd6l, Mef2c, Meis3, Tead4, and Runx1.

**Conclusions:**

The results indicate that the extensive alterations in DNA methylation patterns along the development of neonatal murine hearts are likely to contribute to the decline of regenerative capabilities observed shortly after birth. This conclusion is supported by the evidence that an increase in DNA methylation in the neonatal murine heart from day 1 to day 7 occurs in the promoter regions of genes playing important roles in cardiovascular system development.

**Electronic supplementary material:**

The online version of this article (doi:10.1186/s12864-016-2545-1) contains supplementary material, which is available to authorized users.

## Background

The neonatal heart possesses a temporary robust regeneration potential [1–3]. The partial apex resection made in the hearts of 1 day-old, but not in 7-day-old murine neonates, were shown to heal completely within 21 days [1]. The observed regeneration process is driven by the division of pre-existing cardiomyocytes [1, 2], though the stem cells have been reported to contribute to heart regeneration to some extent [4]. Unfortunately, the rate at which cardiomyocytes divide is very low and it is gradually reduced with age [5]. The mechanisms that restrict cardiomyocytes ability to divide in response to injury shortly after birth are poorly understood. One of the triggers that contribute to the cardiomyocyte cell cycle arrest is the transfer from the hypoxic to normal oxygen conditions [6]. The cell cycle arrest has also been linked with the up-regulation of multiple members of the miR-15 family of microRNA, that regulate a number of cell cycle genes [7].

Another important heart regeneration regulator is the Meis1 homeodomain transcription factor which targets several cycline-dependent kinase inhibitors [8]. Additionally, the neonatal heart regeneration capacity has been connected with the Yap protein, a Hippo signalling pathway transcriptional co-factor, that is essential for proper heart development as it regulates insulin growth factor and WNT signalling pathways [9]. Moreover, the changes in heart regeneration capacity have been associated with the maturation of immune system, in particular, with a macrophage population existing in the heart of neonatal mice [10].

Exploring the mechanisms involved in heart development appears to be crucial in designing a scheme for the successful stimulation of heart regeneration. Among the gene regulation mechanisms, DNA methylation re-patterning plays an essential role in development, tissue differentiation, and cell specialisation. In vertebrates, DNA methylation is primarily observed as a mechanism in which a methyl group is added to cytosines within CpG dinucleotides by DNA methyltranferases. DNA methylation and demethylation processes together with histone modification affect chromatin condensation and accessibility of transcription factor binding sites, thus either blocking or enabling transcriptional activation. DNA methylation at promoter regions is typically associated with transcriptional repression, while that of gene bodies is considered to promote gene expression [11]. In this study, the focus is on DNA methylation changes that occur in the murine neonatal heart within the first week of life, between the first day of life when the heart is able to regenerate and the seventh day after birth, when this ability to regenerate is lost.

## Results

### Differentially methylated regions

In order to identify the genomic regions that change their DNA methylation status in murine hearts shortly after birth, we used the methylated DNA immunoprecipitation (MeDIP) approach followed by hybridization with the promoter microarray platform (Mouse DNA Methylation 3x720K CpG Island Plus RefSeq Promoter Array). This platform interrogates 15,980 CpG Islands and 20,404 promoter regions corresponding to 22,881 transcripts.

**Fig. 1:**
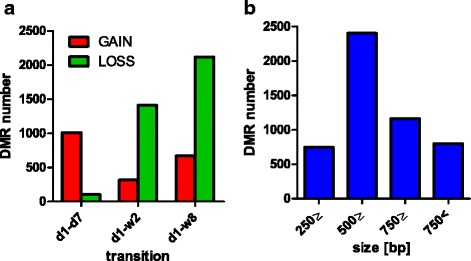
The numbers of differentially methylated DNA regions (DMRs) in murine neonatal hearts. **a** The numbers of DMRs between d1 and d7, w2 and w8. **b** The distribution of the DMRs’ sizes

DNA methylation profiles in the whole hearts of 1-day (d1) neonates were obtained and compared with those of 7-day (d7), 2-week (w2) and 8-week (w8) old mice. Approximately 5000 differentially methylated genomic regions (DMRs) were determined (Fig. 1) and mapped to 4274 genes.

This number of DMRs includes 1113 genomic regions where significant changes in DNA methylation levels were found between d1 and d7, which is to say, before and after the loss of transient regenerative capacity in the neonatal murine heart. 1009 of these regions corresponding to 929 genes were found to show an increase in DNA methylation at d7 (Fig. 1a).

Sixty of these regions corresponding to 55 genes retained an altered DNA methylation status until adulthood (w8) (Table 1). Forty two of these genes showed an increase of DNA methylation following d1. The complete lists of identified DMRs with their chromosome localisations and mapped annotations are included in Additional file 1: F1.

**Table 1:**
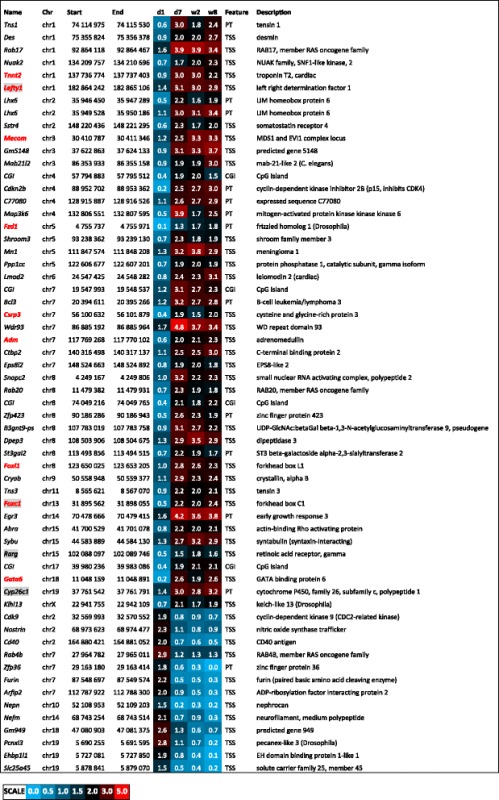
DNA methylation changes in murine hearts following day 1 after birth and retained till adulthood

ᅟ

The locations of genomic regions showing DNA methylation changes in murine hearts between the day 1, 7, and 2 weeks after birth, and retained till adulthood in 8-week-old mice. The genes involved in heart development are marked with red font while those participating in anterior/posterior pattern specification are distinguished by grey shading

*CGI* CpG genomic island; the genomic coordinates listed are mapped to NCBI37/mm9 build

**Fig. 2:**
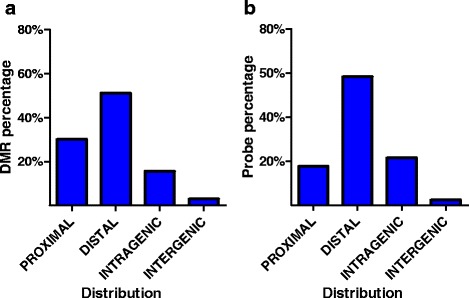
Genome distribution of differentially methylated regions along the development of murine hearts. **a** Genome distribution of the DMRs: proximal - +500: -100 bp from TSS, distal - +5000 : +500 bp from TSS; intragenic - associated with a CpG island located within a primary transcript; intergenic - located over 5000 bp upstream and over 500 bp downstream of any of primary transcripts in the analysis; **b** Probe distribution on the microarray

The majority of changes we found within the first week of life involved the gains of DNA methylation, while for those identified between d1 and w2, as well as, d1 and w8, the loss in DNA methylation is more prevalent (Fig. 1a). Furthermore, multiple genomic regions showed a transient gain and loss of DNA methylation. This indicates the dynamism of epigenetic reprograming in the murine heart within two weeks after birth. The majority of the identified DMRs are between 500 and 750 bp in length (Fig. 1b) and they are located more than 500 bp upstream of their transcription start sites (Fig. 2a). The distributions of DMR locations largely correspond to array design (Fig. 2b). As the majority of hybridization probes in the array correspond to the nucleotide sequences in the vicinity of transcription start sites, a prevailing number of DMRs were mapped to promoter regions (Fig. 2a).

### Gene ontology analysis for genes mapped to differentially methylated regions

**Fig. 3:**
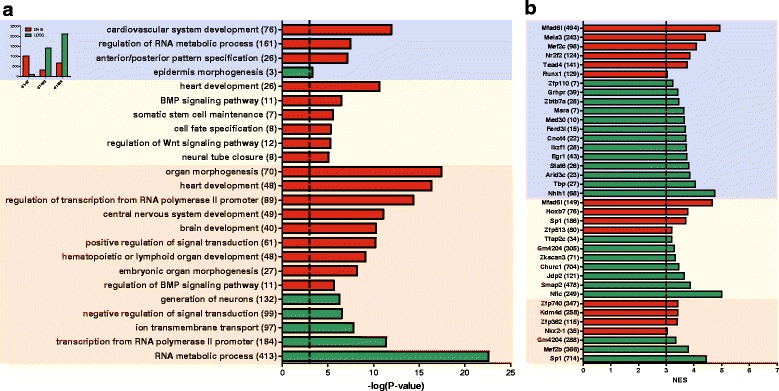
Ontology analysis of genes mapped to genomic regions differentially methylated during development of murine heart. **a** Ontological terms associated with the genes overrepresented among the genes mapped to the DMRs between d1 and d7 (blue), d1 and w2 (yellow), and d1 and w8 (red). Gene set enrichment analyses were performed and gene ontology annotations were found with ClueGo. The results are presented as -log(*P*-value). All presented *p*-values were lower than 0.05. The numbers of corresponding DMRs are shown in the inset. **b** Transcription factor binding sites enriched for the genes associated with the DMRs between d1 and d7 (blue), d1 and w2 (yellow), and d1 and w8 (red). The analysis was carried out with iRegulon. The results are presented as Normalized Enrichment Scores (NES). The numbers of genes associated with the listed terms/transcription factors are shown in parentheses

**Fig. 4:**
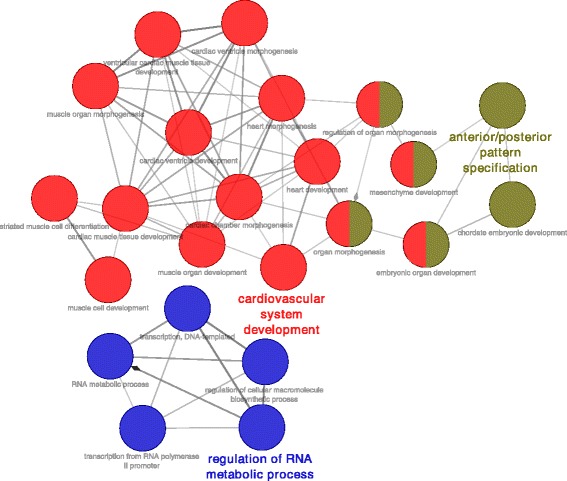
Key ontological categories associated with genes showing an increase in DNA methylation at d7 relative to d1 in neonatal murine hearts. The diagram was generated with ClueGO

A gene ontology analysis, performed with ClueGo, showed a significant overrepresentation of genes associated with heart development for the regions that gain DNA methylation at d7, w2 and w8 relative to d1 (Fig. 3a). What is more, for the genes associated with the DMRs between d1 and w2, the significant functional categories include the regulation of Wnt and BMP signalling, as well as the maintenance of somatic stem cells. With regard to revealing potential regulators associated with the epigenetic transition, a prediction of transcriptional factors targeting the differentially methylated genes was carried out (Fig. 3b). The functional terms connected with the DNA methylation increase between d1 and d7 were clustered into three main categories: cardiovascular system development, anterior/posterior patterns formation, and regulation of RNA metabolic process (Fig. 4).

The DMRs between d1 and d7 show a set of 60 changes in DNA methylation which are retained until w8 (Table 1). These 60 DMRs are enriched in genes involved with cardiovascular system development. The group of genes includes those of sarcomere proteins (*Des*, *Tnnt2*), transcription regulators (*Gata6, Ctbp2*), and developmental proteins (*Lefty1, Fzd1*).

The complete results of gene ontology analyses are included in Additional file 1: F1.

### Prediction of transcriptional factors targeting differentially methylated genes

As determined with iRegulon analysis, the promoter regions of genes associated with the gain of DNA methylation at d7 relative to d1 show a remarkable enrichment with the sequence motifs of Mfsd6l, a transcriptional factor which targets 494 out of the 929 genes which display an increase in DNA methylation level at d7 as compared to d1. Further enriched motifs are related to a cardiac specific myocyte enhancer factor Mef2c, Meis3 homeobox, Tead4 transcription factor, and the runt-related transcription factor Runx1. The promoter regions of genes which display a decrease in DNA methylation at d7 vs. d1 show an enrichment for the motifs associated with Stat6, a regulator of Il4 and Il13 signalling, which are known to play important roles in immune responses (Fig. 3b).

The complete results of iRegulon predictions are included in Additional file 1: F1.

### Differentially expressed genes

With a view to investigating the alterations in transcriptome profiles which accompany those of methylome, genome-wide gene expression profiling in the neonatal and adult murine heart tissues was performed using Mouse Gene Expression 12x135K microarray. The array interrogates 44,170 transcripts corresponding to over 24,200 genes. Additionally, transcriptome profiling of embryonic heart tissues was performed in order to identify the genes which show similar expression profiles in embryos and d1 neonates and that are greatly up- or down-regulated in the further phases of development and in adults.

**Fig. 5:**
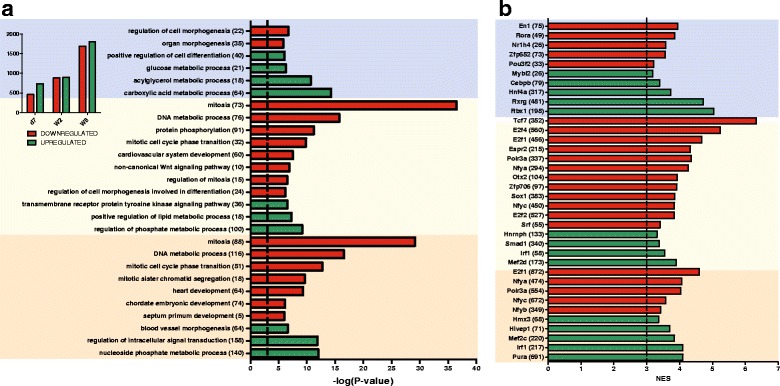
Ontology analysis of genes differentially expressed during development of murine heart. **a** The ontological terms were revealed by gene set enrichment analysis for the genes showing at least a two-fold change in expression at d7 (blue), w2 (yellow) and w8 (red) in comparison to d1. Gene set enrichment analyses were performed and gene ontology annotations were found with ClueGo. The results are presented as -log(*P*-value). The numbers of differentially regulated genes are shown in the inset. **b** Transcription factor binding sites enriched for the differentially regulated genes between d1 and d7 (blue), d1 and w2 (yellow), and d1 and w8 (red). The analysis was carried out with iRegulon. The results are presented as Normalized Enrichment Scores (NES). The numbers of genes associated with the listed terms/transcription factors are shown in parentheses

We singled out the genes showing at least a two-fold change in expression between d1 and d7 and between w2 and w8; these genes are further referred to as “differentially expressed”. An ontology analysis performed with ClueGO shows that a significant group of genes differentially expressed between d1 and d7 are associated with the regulation of cell morphogenesis (up-regulated at d1) and glucose metabolism (down-regulated at d1), while among those displaying a two-fold change between d1 and w2, as well as between d1 and w8, there are distinguishing groups connected with the regulation of mitosis and heart development (Fig. 5a). The genes that show at least a two-fold change in expression observed in the first week of life and are retained until adulthood are shown in Table 2. The prediction of transcription factors targeting the differentially expressed genes is presented in Fig. 5b.

**Table 2:**
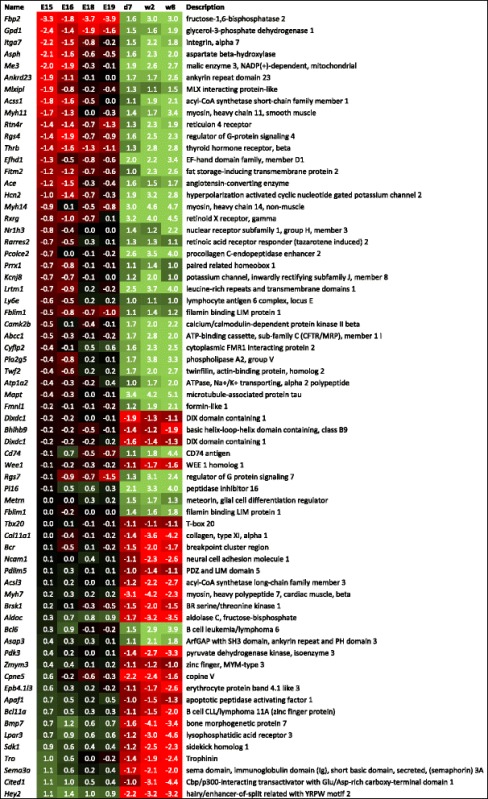
Gene expression changes in murine hearts following d1 after birth, and retained till adulthood

ᅟ

The genes which display at least a two-fold change in expression levels in murine hearts between the day 1 and 7 after birth, which is retained in 2-week-old and till adulthood in 8-week old mice. The results are presented as log2 ratios of normalized microarray signals relative to d1

### Changes in DNA methylation and gene expression

**Fig. 6:**
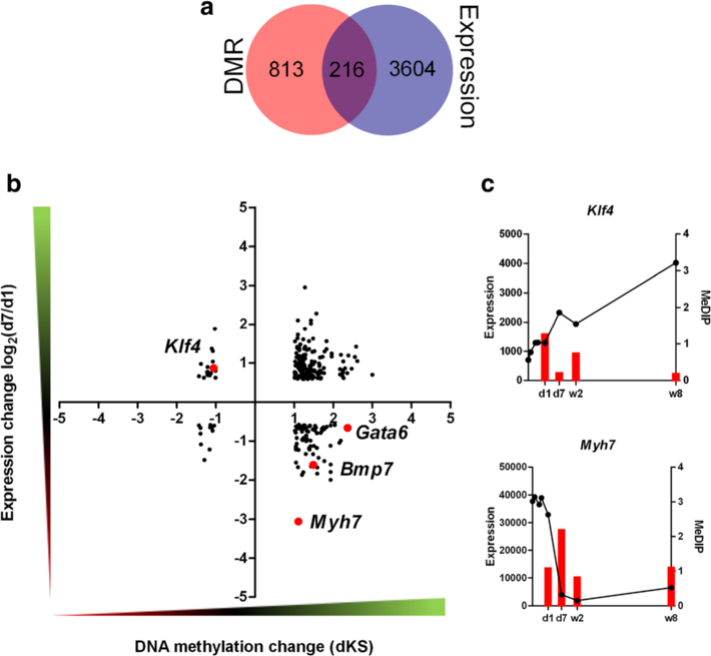
DNA methylation and gene expression changes in neonatal murine hearts between d1 and d7. **a** The number of genes associated with the DMRs between d1 and d7 and those showing at least a 1.5-fold change in expression between these time-points (**b**) Dot plot representation (**c**) and representative examples of genes changing the expression and DNA methylation status between d1 and d7. The numbers of dots representing the genes are listed in Table 3. The complete lists of genes are included in Additional file 1: F1

**Fig. 7:**
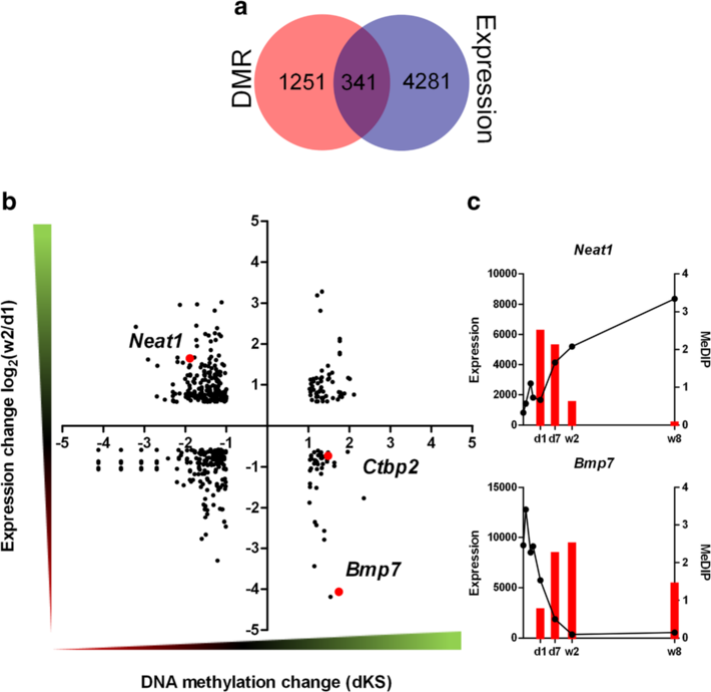
DNA methylation and gene expression changes in murine hearts between the d1 and 2 weeks. **a** The number of genes associated with the DMRs between d1 and w2 and those showing at least a 1.5-fold change in expression between these time-points (**b**) Dot plot representation (**c**) and representative examples of genes changing the expression and DNA methylation status between d1 and w2. The numbers of dots representing the genes are listed in Table 3. The complete lists of genes are included in Additional file 1: F1

**Fig. 8:**
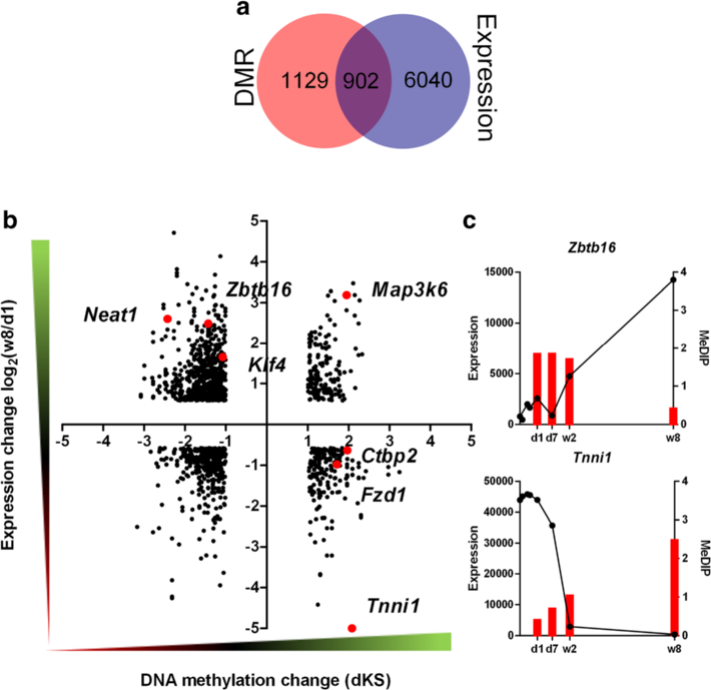
DNA methylation and gene expression changes in murine hearts between d1 newborns and adults. **a** The number of genes associated with the DMRs between d1 and w8 and those showing at least a 1.5-fold change in expression between these time-points (**b**) Dot plot representation (**c**) and representative examples of genes changing the expression and DNA methylation status between d1 and w8. The numbers of dots representing the genes are listed in Table 3. The complete lists of genes are included in Additional file 1: F1

As a rule, increased DNA methylation of promoter regions is correlated with a decrease in the level of gene expression. However, an increase of DNA methylation within gene bodies may promote transcription [11]. In order to investigate as to whether the alterations of gene methylation profiles in murine hearts observed after birth are reflected in gene expression, we carried out a search for the correlations between the DNA methylation and gene expression changes. In this purpose, we paired the genes changing expression by at least 1.5-fold with their corresponding DMRs. As the result, we identified 216, 341, and 902 genes for which the change of expression can be associated with the DMRs between d1 and d7, d1 and w2, and d1 and w8 hearts, respectively (Table 3). The relationships between DNA methylation and gene expression changes are demonstrated using coordinate plots, where the dots in the 1^st^ and the 3^rd^ quadrants represent the positive correlations_,_ while those in the 2^nd^ and the 4^th^ quadrants, the inverse ones (Figs. 6, 7, 8). Apart from the expected inverse correlations between DNA methylation and gene expression, we identified comparable numbers of the positive ones. There are, however, a relatively small fraction of genes in the analysis that displayed correlations, either positive or negative, between DNA methylation and gene expression changes, as shown by the Venn diagrams in Figs. 6, 7, 8. The genes showing transcriptional down-regulation at d1 vs. d7 accompanied by a gain in DNA methylation were significantly enriched in those involved in heart development. No significant ontological categories were determined for the genes displaying transcriptional up-regulation correlated with a decreased DNA methylation at d1 vs. d7. Surprisingly, several developmental genes reported to participate in heart functions, showed an increase in DNA methylation and expression at d7 vs. d1 (Fig. 6, Table 4).

**Table 3:** Numbers and vectors of DNA methylation and gene expression changes in murine hearts after birth

		Number of DMRs and transcripts pairs			
	Relationship	Distal	Proximal	Intragenic	Total
d1-d7	MeDIP↑ Expression↑	89	43	28	160
	MeDIP↑ Expression↓	55	17	11	83
	MeDIP↓ Expression↑	6	6	6	18
	MeDIP↓ Expression↓	6	5	1	12
d1-w2	MeDIP↑ Expression↑	21	17	19	57
	MeDIP↑ Expression↓	14	21	9	44
	MeDIP↓ Expression↑	146	30	50	226
	MeDIP↓ Expression↓	27	119	44	190
d1-w8	MeDIP↑ Expression↑	121	23	23	167
	MeDIP↑ Expression↓	143	43	13	199
	MeDIP↓ Expression↑	245	311	118	674
	MeDIP↓ Expression↓	135	178	97	410

The summary includes the numbers of DMRs paired with corresponding transcripts changing their expression levels by at least a 1.5-fold. The relationships are visualized in Figs. 6, 7, 8

MeDIP↑/MeDIP↓ - DNA methylation gain/loss

Expression↑/Expression↓ - increase/decrease

**Table 4:** Significant functional terms found for the genes displaying an increase in DNA methylation and expression at d7 relative to d1 in neonatal murine hearts

GOID	GOTerm	*p*-value corrected with Bonferroni step down	Associated Genes Found
GO:0098742	cell-cell adhesion via plasma-membrane adhesion molecules	8.09E-04	*Cdh1, Cdh13, Cdh26, Esam, Igsf9, Pcdhga12, Scarf2, Umod*
GO:0048705	skeletal system morphogenesis	1.43E-03	*Alpl,* ***Bmp4*** *, Chad, Hoxa3, Hoxa5, Hoxb5, Ltbp3,* ***Osr1*** *, Tcf15,* ***Tgfb3***
GO:0009952	anterior/posterior pattern specification	5.37E-03	***Bmp4*** *, Hoxa3, Hoxa5, Hoxb5, Med12, Meox1,* ***Osr1*** *, Tcf15,* ***Zic3***
GO:0031032	actomyosin structure organization	1.01E-02	*Ankrd23, Asap3,* ***Lmod2*** *,* ***Myh7b, Mypn*** *,* ***Neb*** *,* ***Tpm1***
GO:0048771	tissue remodelling	1.68E-02	*Clec10a,* ***Dll4*** *, Hoxa3, Ltbp3, Nol3, Pml, Spp2*
GO:0055002	striated muscle cell development	3.74E-02	*Ankrd23,* ***Bmp4, Lmod2, Mypn, Neb*** *, P2rx2*
GO:0045064	T-helper 2 cell differentiation	8.02E-03	*Bcl3, Hlx, Irf1*
GO:0005520	insulin-like growth factor binding	3.24E-02	*Cyr61, Htra4, Igfbp6*

As revealed by ClueGo gene ontology analysis, a group of 215 genes showing a gain in DNA methylation and a minimum 1.5-fold increase in expression level was significantly enriched in developmental genes. Remarkable genes reported to participate in heart functions a development are distinguished by bold font. (The 215 genes are are listed in Additional file 1: F1 quadrant of Fig. 8)

### Validation of microarray results by using quantitative-PCR

**Fig. 9:**
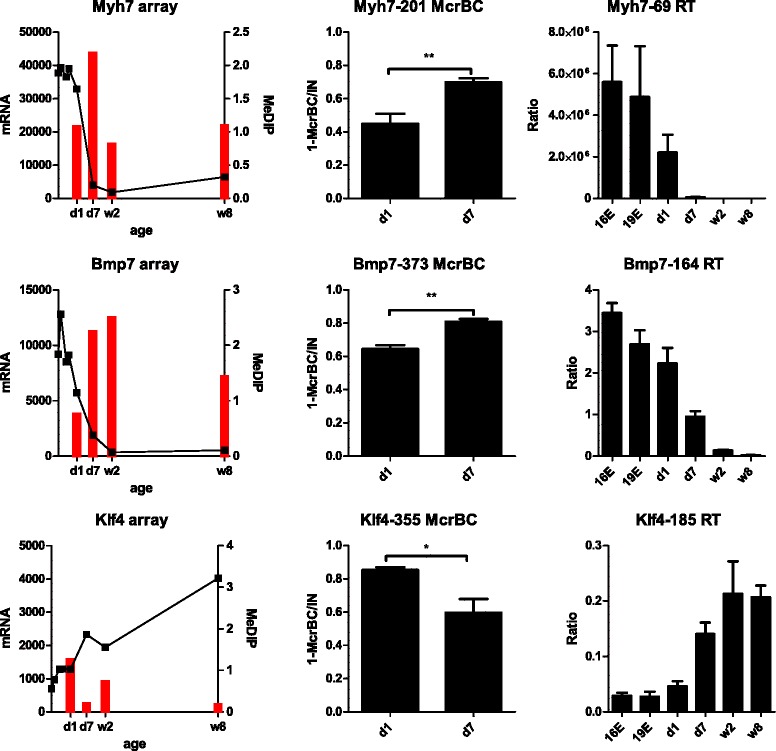
qPCR validation of microarray results for remarkable genes showing significant changes in DNA methylation and transcript levels between d1 and d7 in neonatal murine hearts. The left panels show DNA methylation and gene expression microarray results represented by red bars and black markers, respectively; DNA methylation is expressed as MeDIP enrichment and gene expression levels as normalized microarray signals. The black markers preceding that indicating the neonatal d1 represent gene expression levels in embryonic hearts E16, E18, E19, E20. The middle panels present CpG methylation estimated with Methylation Dependent Restriction Digestion followed by quantitative PCR (MDRE-qPCR) where the DNA methylation levels correspond to the amounts of DNA undigested by CpG methylation dependent McrBC enzyme (1-(McrBC/Input). The right panels demonstrate transcript levels determined with qPCR as the ratios to the reference transcript of the *Tbp* gene (TATA binding protein gene). The microarray results were determined for pooled samples of 3 mice. The qPCR results represent average values obtained for three individuals for each developmental time-point. The statistical significance has been determined with two-tailed heteroscedastic Student’s *t*-test. The complete results of qPCR validation are collected in Additional file 2: F2

The DNA methylation and gene expression microarray results were validated for a selection of transcripts and DMRs using qPCR as a reference method. DNA methylation levels were estimated by DNA digestion with a CpG methylation dependent McrBC enzyme followed by qPCR quantitation. Gene expression levels were determined by using qPCR with primers targeting the transcript regions corresponding to the microarray probes. Representative examples of qPCR validations are shown in Fig. 9 and the complete results are collected in Additional file 2: F2. The qPCR quantitation confirmed the microarray results.

## Disscussion

It has been recently shown that murine hearts undergo dynamic DNA methylation reprogramming after birth, with waves of global DNA demethylation and methylation. It has been reported that 5-azacytidine treatment increases cardiomyocyte proliferation in the neonatal heart and it affects cardiomyocyte binucleation [12]. What is more, the epigenetic pattern in the cardiomyocytes from the adult injured heart changes to become similar to that in neonates [13]. The transcriptional analysis of neonatal ventricles after injury showed a reversion of cardiomyocyte transcription profiles towards a less differentiated state [14].

The study by Sim et al. [12] compares global DNA methylation profiles of heart left ventricles obtained by the enrichment of methylated DNA with methyl-CpG-binding domain followed by DNA sequencing (MBD-seq) between 1-day- and 2-week-old mice. The article by Gilsbach et al. [13] reports the examination of DNA methylation profiles in isolated cardiomyocytes from neonatal (1-day-old) and adult (8-week-old) mice performed by genome bisulphite sequencing.

In our study, the changes in the postnatal global DNA methylation profiles in the whole murine hearts were examined by using methylated DNA immunoprecipitation (MeDIP) followed by microarray analysis targeting promoter regions. We compared DNA methylation profiles in the hearts of 1-day-old newborns with those of 7-day-, 2-week-, and 8-week-old mice. Since the neonatal heart loses its regeneration capacity within the first week of life, we focused the analysis on DNA methylation changes that occur between the days 1 and 7.

The number of DNA methylation gains exceeds that of losses at d7 relative to d1, while the proportion is reversed for the comparison between d1 and w2 and that between d1 and w8. A significant number of genes among those associated with the genomic regions showing an increase in DNA methylation from d1 to d7 are known to participate in heart development. As indicated by bioinformatics tools, the genes exhibiting an increased methylation in the 7-day-old relative to 1-day-old neonates are enriched in the targets of several transcriptional regulators: Mef2c, Nr2f2, Tead4, Meis3, and Mfsd6l. The first three factors are known for their roles in heart functions and defects. The cardiac specific muscle enhancer factor Mef2c together with Gata4 and Tbx5, has been reported to reprogram neonatal fibroblast into cardiomyocyte-like cells [15]. The nuclear receptor subfamily 2, group F, member 2, Nr2f2, has been associated with congenital heart defects [16] and tetralogy of Fallot [17] in humans. The TEA domain family member 4, Tead4 has been demonstrated to induce hypertrophy of rat cardiomyocytes through α_1_-adrenergic receptor stimulation [18] and to up-regulate *Hif1α,* thus stimulating vascular development and heart recovery after ischemia [19]. Meis3 and Mfsd6l have not been reported in the context of heart development to date but, as indicated by the above mentioned bioinformatics prediction, they are likely to target 243 and 494, respectively, of 929 genes mapped to the DMRs hypomethylated at d1 relative to d7. Nevertheless, the predicted transcriptional factors targeting the differentially methylated and those targeting the differentially expressed genes largely do not overlap (Fig. 3b and Fig. 5b). However, we were able to select several genes targeted by the transcriptional regulators Mef2c, Nr2f2, Tead4, Meis3, and Mfsd6l that display a decrease in expression correlated with a gain in DNA methylation at d7 as compared to d1 (Additional file 3: F3).

A number of differentially methylated genes show inverse correlations between the changes in DNA methylation and transcription levels observed from d1 to d7 (Figs. 6, 7, 8). The group includes the *Klf4* and *Bmp7* genes, which encode regulatory factors, and *Myh7*, that encodes the heavy subunit of cardiac myosin (Fig. 9). It has been reported that cardiomyocyte-specific *Klf4* knock-out mice display an exaggerated expression of cardiac foetal genes after induced cardiac hyperthrophy [20] and the endothelial *Klf4* is up-regulated after the loss of cerebral cavernous malformation signalling, which results in heart failure [21]. *Bmp7* together with *Bmp6* play a role in the formation of cardiac cushions [22]. *Bmp7* inhibits endothelial-mesenchymal transition, as well as cardiac fibrosis after heart injury [23].

Multiple other genes show inverse correlations between promoter DNA methylation and transcript levels changes in the course of heart development (Figs. 7, 8). Representative examples of such genes include: *Neat1, Zbtb16*, *Tnni1*, and *Fzd1. Neat1* and *Zbtb16* show an increase in expression correlated with a decrease in DNA methylation along the development (Additional file 2: F2). *Neat1* is a gene of long non-coding RNA which acts as a core structural component of nuclear paraspeckles. *Neat1* is expressed in various tissues and it has been reported to protect the heart from pathological hypertrophy [24]. *Zbtb16* encodes a transcription factor associated with cell cycle progression. *Tnni1 and Fzd1* display decreasing expression connected with increasing DNA methylation within heart development (Additional file 2: F2). *Tnni1* is a gene of a foetal muscle troponin [25]. *Fzd1* encodes a receptor of WNT signalling, which plays an important role in heart development and in heart tissue remodelling under pathological conditions [26].

We found that a number of genes exhibited positive correlations between DNA methylation and gene expression changes between d1 and d7 (Fig. 6). We observed an increase in promoter DNA methylation and expression for a remarkable group of genes participating in heart functions and development such as *Myh7b* and *Bmp4* (Table 4). This observation could be explained by cell specialisation in the growing hearts.

It is also important to stress that in this research DNA and RNA were extracted from whole hearts, therefore the observed differences pertain to mixed cell populations including cardiomyocytes, fibroblast and endothelial cells. The limited ability of cardiomyocytes to proliferate shifts the focus of heart regeneration studies on this type of cells. However, the involvement of cardiac fibroblasts is also worth exploring as the secretory functions of fibroblasts and their role in scar formation should be taken into consideration.

## Conclusions

This may be considered the first report to show the changes in DNA methylation profiles in the neonatal murine hearts between day 1 and day 7 points in time which delineate the transient regenerative ability of neonatal murine hearts. The results indicate a number of differentially methylated regions between day 1 and day 7, most of them increasing DNA methylation at day 7. The DMRs are significantly enriched in genes associated with muscle development and embryonic morphogenesis that are also critical for proper heart maturation. The results of this study indicate a group of transcriptional regulators which target the genes displaying decreased methylation at day 1 vs. day 7. Three of them Mef2c, Nr2f2, Tead4 are known to participate in heart development and functions, thus suggesting their involvement in the regenerative repair in neonatal hearts. Two other regulators: Mfsd6l and Meis3, are not known to have been reported in the context of the heart. Our findings show that DNA methylation changes, largely an increase in gene methylation, is accompanied by a decrease of heart regenerative ability in the first week of life in neonatal mice. In addition, the results indicate candidate genes and transcription factors involved in the process.

## Methods

### Tissue samples and nucleic acid extraction

The hearts of C57BL/6J mice at the age of 8 weeks (w8) were purchased from the Jackson Laboratories (Bar Harbor, USA). The hearts of the C57BL/6J embryos (embryonic day 15, 16, 18 and 19 (E15, E16, E18, E19) and murine neonates at the age of 1 day (d1), 7 days (d7) and 2 weeks (w2) were purchased from the Tri-city Experimental Animal Centre - Research and Services Centre - Medical University of Gdańsk (Poland). The tissues were collected on RNA Later (Qiagen, cat. no. 76104) and transported in dry ice. Tissues were disrupted with mortar and pestle in liquid nitrogen and divided into two portions, for RNA and DNA isolation. Genomic DNA was extracted by using DNeasy Blood and Tissue Mini Kit (Qiagen, cat. no. 69504) with RNaseA treatment (Qiagen, cat. no. 19101) for RNA removal. Total RNA was extracted with RNeasy Mini Kit (Qiagen, cat. no. 74104) with on-column DNA digestion with RNase-Free DNase Set Kit (Qiagen, cat. no. 79254).

### DNA methylation profiling

Methylated DNA immunoprecipitation (MeDIP) followed by microarray analysis was performed for tissue pools collected from three individuals at d1, d7, w2 and w8. MeDIP, labelling, hybridization and image acquisition were performed as previously described [27] using Mouse DNA Methylation 3x720K CpG Island Plus RefSeq Promoter Arrays (Roche, NimbleGen).

Image acquisition was done with an MS 200 Scanner (Roche, NimbleGen) at 2 μm resolution by using high-sensitivity auto-gain settings. Data processing was performed with NimbleScan v. 2.6 (Roche, NimbleGen), which included obtaining raw data files, normalizing the raw data by using quantile normalization with background correction separately for each channel, following computing biweighted log_2_ ratios, determining KS Scores (with 750 bp sliding window and 500 bp spacing between nearby probes) and mapping DNA probes to genes and CpG islands using mm9/NCBI37 build. The data files have been deposited in Gene Expression Omnibus Database under the accession number GSE68524.

### Gene expression microarray profiling

Gene expression profiling was performed for embryonic murine hearts (E15, E16, E18, E19) in addition to those of d1, d7, w2 and w8 mice. Total RNA from three individuals for each time-point were pooled in equal amounts and 3 μg of RNA was used for cDNA synthesis. The first strand cDNA was synthesized with 200 units of Maxima Reverse Transcriptase (ThermoScientificBio, cat. no. EP0742) and the second strand with cDNA Synthesis System (Roche, cat. no. 11 117 831 001) according to the manufacturer’s protocol. The double stranded cDNA samples were labelled with Cy3 using a NimbleGen One-Color DNA labelling kit (Roche, cat. no. 06370411001) and hybridized to NimbleGen Mouse Gene Expression 12x135K array (Roche, cat. no. 05543797001). Image acquisition was done as described above. Raw data was processed and normalized using a robust multi-chip average (RMA) algorithm using NimbleScan v. 2.6 software with default settings (Nimblegen, Roche). The gene expression data were filtered to remove the results with SE values lower than 0.8 (Additional file 1: F1). The data files have been deposited in Gene Expression Omnibus Database under the accession number GSE68524.

### Identification of differentially methylated regions (DMRs)

The regions that change their methylation after birth were identified by comparing probe enrichment between d1 and the later time-points. A region was considered as a differentially methylated (DMR) if it was delineated by at least three consecutive probes displaying a minimum two-fold increase and not less than 1.0 KS score difference (ΔKS ≥ 1.0) in two contrasted samples. A DMR is referred as *proximal* if it is situated from +500 bp up to -100 bp from the transcription start site, *distal* if it is located from +5000 bp to +500 bp from the transcription start site, *intragenic* if it is associated with a CpG island located within a primary transcript, and *intergenic* if it is located within a CpG island which was not mapped to any promoter regions or primary transcripts, i.e., located over 5000 bp upstream and over 500 bp downstream of any of primary transcripts in the analysis.

### Validation of CpG methylation

CpG methylation levels were examined by using Methylation Dependent Restriction Digestion followed by quantitative PCR (MDRE-qPCR) with McrBC restriction endonuclease (NEB, cat. no. M0272). Approximately 200 ng of genomic DNA was used for digestion in total volume of 10 μl using 10 units of enzyme for 1 h at 37°C. Digested DNA and undigested control (input) was diluted 5 times and 4 ng of DNA was used for subsequent qPCR reactions. qPCR reactions were performed with FastStart Essential DNA Green Master (Roche, cat. no. 06402712001) in Light Cycler 96 instrument (Roche). The results are presented as 1-(McrBC/Input) in total volume of 10 μl. The primer sequences are listed in Additional file 4: F4.

### Gene expression validation

Gene expression levels were examined with reverse transcription reaction followed by quantitative PCR. Approximately 200 ng of total RNA was used for reverse transcription reactions with Maxima Reverse Transcriptase (ThermoFisher Scientific, cat. no. EP0741) and oligo dT20 primer. The cDNA templates were diluted 5 times and 2 μl of the solution was used for qPCR reactions that were performed with FastStart Essential DNA Green Master (Roche, cat. no. 06402712001) in Light Cycler 96 instrument. The primer sequences are listed in Additional file 4: F4.

### Bioinformatics tools

Gene ontology analysis was performed with ClueGO [28] using right-sided hypergeometric test with Bonferroni step down correction. The sequence motif enrichment and transcription factor predictions were obtained for the genes annotated to DMRs with iRegulon [29] using a 500 bp interval in proximity to transcription start sites.

## Ethical statement

The ethical approval for the collection of murine hearts no. 18/2014 was issued by the Local Ethics Commission for Experimentation on Animals at the Medical University of Gdansk, Poland.

## Availability of data and materials

The raw and normalized genome-wide DNA methylation and gene expression data supporting the results of this article are available in the Gene Expression Omnibus (GEO) repository under the accession number GSE68524 http://www.ncbi.nlm.nih.gov/geo/query/acc.cgi?token=klgvsskqpvsplgn&acc=GSE68524.

## Additional files

Additional file 1: F1. The lists of differentially methylated genomic regions and differentially expressed genes and the complete results of gene ontology analyses. (XLSX 16191 kb) Additional file 2: F2. qPCR validation of microarray results – complete results. The left panels show DNA methylation and gene expression microarray results represented by red bars and black markers, respectively; DNA methylation is expressed as MeDIP enrichment and gene expression levels as normalized microarray signals. The black markers preceding that of neonatal d1 represent gene expression levels in embryonic hearts E16, E18, E19, E20. The middle panels present DNA methylation levels estimated with Methylation Dependent Restriction Digestion followed by qPCR (MDRE-qPCR) where the methylation levels correspond to the amounts of DNA undigested by CpG methylation dependent McrBC enzyme (1-(McrBC/Input). The right panels demonstrate transcript levels determined with qPCR as the ratios to the reference transcript of the *Tbp* gene (TATA binding protein gene). The microarray results were determined for pooled samples of 3 mice. The qPCR results represent average values obtained for three individuals for each developmental time-point. The statistical significance has been determined with two-tailed heteroscedastic Student’s *t*-test. (PDF 33 kb) Additional file 3: F3. The target genes of transcriptional regulators Mef2c, Nr2f2, Tead4, Meis3, and Mfsd6l hypomethylated and up-regulated at d1 vs. d7 in the neonatal murine heart. (XLSX 9 kb) Additional file 4: F4. PCR primers used for qPCR validation of microarray results. MDRE-qPCR – primers used for the estimation of DNA methylation. RT-qPCR – primers used to determine transcript levels. (XLSX 10 kb)

## References

[1] Porrello ER, Mahmoud AI, Simpson E, Hill JA, Richardson JA, Olson EN, Sadek HA (2011). Transient regenerative potential of the neonatal mouse heart. Science.

[2] Porrello ER, Mahmoud AI, Simpson E, Johnson BA, Grinsfelder D, Canseco D, Mammen PP, Rothermel BA, Olson EN, Sadek HA (2013). Regulation of neonatal and adult mammalian heart regeneration by the miR-15 family. Proc Natl Acad Sci U S A.

[3] Porrello ER, Olson EN (2014). A neonatal blueprint for cardiac regeneration. Stem Cell Res.

[4] Hsieh PC, Segers VF, Davis ME, MacGillivray C, Gannon J, Molkentin JD, Robbins J, Lee RT (2007). Evidence from a genetic fate-mapping study that stem cells refresh adult mammalian cardiomyocytes after injury. Nat Med.

[5] Senyo SE, Steinhauser ML, Pizzimenti CL, Yang VK, Cai L, Wang M, Wu TD, Guerquin-Kern JL, Lechene CP, Lee RT (2013). Mammalian heart renewal by pre-existing cardiomyocytes. Nature.

[6] Puente BN, Kimura W, Muralidhar SA, Moon J, Amatruda JF, Phelps KL, Grinsfelder D, Rothermel BA, Chen R, Garcia JA (2014). The oxygen-rich postnatal environment induces cardiomyocyte cell-cycle arrest through DNA damage response. Cell.

[7] Porrello ER, Johnson BA, Aurora AB, Simpson E, Nam YJ, Matkovich SJ, Dorn GW, van Rooij E, Olson EN (2011). MiR-15 family regulates postnatal mitotic arrest of cardiomyocytes. Circ Res.

[8] Mahmoud AI, Kocabas F, Muralidhar SA, Kimura W, Koura AS, Thet S, Porrello ER, Sadek HA (2013). Meis1 regulates postnatal cardiomyocyte cell cycle arrest. Nature.

[9] Xin M, Kim Y, Sutherland LB, Murakami M, Qi X, McAnally J, Porrello ER, Mahmoud AI, Tan W, Shelton JM (2013). Hippo pathway effector Yap promotes cardiac regeneration. Proc Natl Acad Sci U S A.

[10] Aurora AB, Porrello ER, Tan W, Mahmoud AI, Hill JA, Bassel-Duby R, Sadek HA, Olson EN (2014). Macrophages are required for neonatal heart regeneration. J Clin Invest.

[11] Portela A, Esteller M (2010). Epigenetic modifications and human disease. Nat Biotechnol.

[12] Sim CB, Ziemann M, Kaspi A, Harikrishnan KN, Ooi J, Khurana I, Chang L, Hudson JE, El-Osta A, Porrello ER (2014). Dynamic changes in the cardiac methylome during postnatal development. Faseb J.

[13] Gilsbach R, Preissl S, Gruning BA, Schnick T, Burger L, Benes V, Wurch A, Bonisch U, Gunther S, Backofen R (2014). Dynamic DNA methylation orchestrates cardiomyocyte development, maturation and disease. Nat Commun.

[14] O'Meara C, Wamstad JA, Gladstone R, Fomovsky G, Butty V, Shrikumar A, Gannon J, Boyer L, Lee RT (2015). Transcriptional reversion of cardiac myocyte fate during mammalian cardiac regeneration. Circ Res.

[15] Ieda M, Fu JD, Delgado-Olguin P, Vedantham V, Hayashi Y, Bruneau BG, Srivastava D (2010). Direct reprogramming of fibroblasts into functional cardiomyocytes by defined factors. Cell.

[16] Nakamura E, Makita Y, Okamoto T, Nagaya K, Hayashi T, Sugimoto M, Manabe H, Taketazu G, Kajino H, Fujieda K (2011). 5.78 Mb terminal deletion of chromosome 15q in a girl, evaluation of NR2F2 as candidate gene for congenital heart defects. Eur J Med Genet.

[17] Sheng W, Qian Y, Zhang P, Wu Y, Wang H, Ma X, Chen L, Ma D, Huang G (2014). Association of promoter methylation statuses of congenital heart defect candidate genes with Tetralogy of Fallot. J Transl Med.

[18] Stewart AF, Suzow J, Kubota T, Ueyama T, Chen HH (1998). Transcription factor RTEF-1 mediates alpha1-adrenergic reactivation of the fetal gene program in cardiac myocytes. Circ Res.

[19] Jin Y, Wu J, Song X, Song Q, Cully BL, Messmer-Blust A, Xu M, Foo SY, Rosenzweig A, Li J (2011). RTEF-1, an upstream gene of hypoxia-inducible factor-1alpha, accelerates recovery from ischemia. J Biol Chem.

[20] Yoshida T, Yamashita M, Horimai C, Hayashi M (2014). Kruppel-like factor 4 protein regulates isoproterenol-induced cardiac hypertrophy by modulating myocardin expression and activity. J Biol Chem.

[21] Zhou Z, Rawnsley DR, Goddard LM, Pan W, Cao XJ, Jakus Z, Zheng H, Yang J, Arthur JS, Whitehead KJ (2015). The cerebral cavernous malformation pathway controls cardiac development via regulation of endocardial MEKK3 signaling and KLF expression. Dev Cell.

[22] Kim RY, Robertson EJ, Solloway MJ (2001). Bmp6 and Bmp7 are required for cushion formation and septation in the developing mouse heart. Dev Biol.

[23] Zeisberg EM, Tarnavski O, Zeisberg M, Dorfman AL, McMullen JR, Gustafsson E, Chandraker A, Yuan X, Pu WT, Roberts AB (2007). Endothelial-to-mesenchymal transition contributes to cardiac fibrosis. Nat Med.

[24] Han P, Li W, Lin C-H, Yang J, Shang C, Nuernberg ST, Jin KK, Xu W, Lin C-Y, Lin C-J. A long noncoding RNA protects the heart from pathological hypertrophy. Nature 2014;514(7520):102–106. 10.1038/nature13596 PMC4184960 25119045

[25] Bedada FB, Chan SS, Metzger SK, Zhang L, Zhang J, Garry DJ, Kamp TJ, Kyba M, Metzger JM (2014). Acquisition of a quantitative, stoichiometrically conserved ratiometric marker of maturation status in stem cell-derived cardiac myocytes. Stem Cell Reports.

[26] Brade T, Männer J, Kühl M (2006). The role of Wnt signalling in cardiac development and tissue remodelling in the mature heart. Cardiovasc Res.

[27] Gornikiewicz B, Ronowicz A, Podolak J, Madanecki P, Stanislawska-Sachadyn A, Sachadyn P (2013). Epigenetic basis of regeneration: analysis of genomic DNA methylation profiles in the MRL/MpJ mouse. DNA Res.

[28] Bindea G, Mlecnik B, Hackl H, Charoentong P, Tosolini M, Kirilovsky A, Fridman WH, Pages F, Trajanoski Z, Galon J (2009). ClueGO: a Cytoscape plug-in to decipher functionally grouped gene ontology and pathway annotation networks. Bioinformatics.

[29] Janky R, Verfaillie A, Imrichova H, Van de Sande B, Standaert L, Christiaens V, Hulselmans G, Herten K, Naval Sanchez M, Potier D (2014). iRegulon: from a gene list to a gene regulatory network using large motif and track collections. PLoS Comput Biol.

