# Supplementary material for: Changes in gene methylation patterns in neonatal murine hearts: Implications for the regenerative potential
Source: BMC Genomics. 2016 Mar 15;17:231. doi: 10.1186/s12864-016-2545-1 (PMC4791959; doi:10.1186/s12864-016-2545-1)
Supplement: Additional file 2: F2. — qPCR validation of microarray results – complete results. The left panels show DNA methylation and gene expression microarray results represented by red bars and black markers, respectively; DNA methylation is expressed as MeDIP enrichment and gene expression levels as normalized microarray signals. The black markers preceding that of neonatal d1 represent gene expression levels in embryonic hearts E16, E18, E19, E20. The middle panels present DNA methylation levels estimated with Methylation Dependent Restriction Digestion followed by qPCR (MDRE-qPCR) where the methylation levels correspond to the amounts of DNA undigested by CpG methylation dependent McrBC enzyme (1-(McrBC/Input). The right panels demonstrate transcript levels determined with qPCR as the ratios to the reference transcript of the Tbp gene (TATA binding protein gene). The microarray results were determined for pooled samples of 3 mice. The qPCR results represent average values obtained for three individuals for each developmental time-point. The statistical significance has been determined with two-tailed heteroscedastic Student’s t-test. (PDF 33 kb) [file 12864_2016_2545_MOESM2_ESM.pdf]

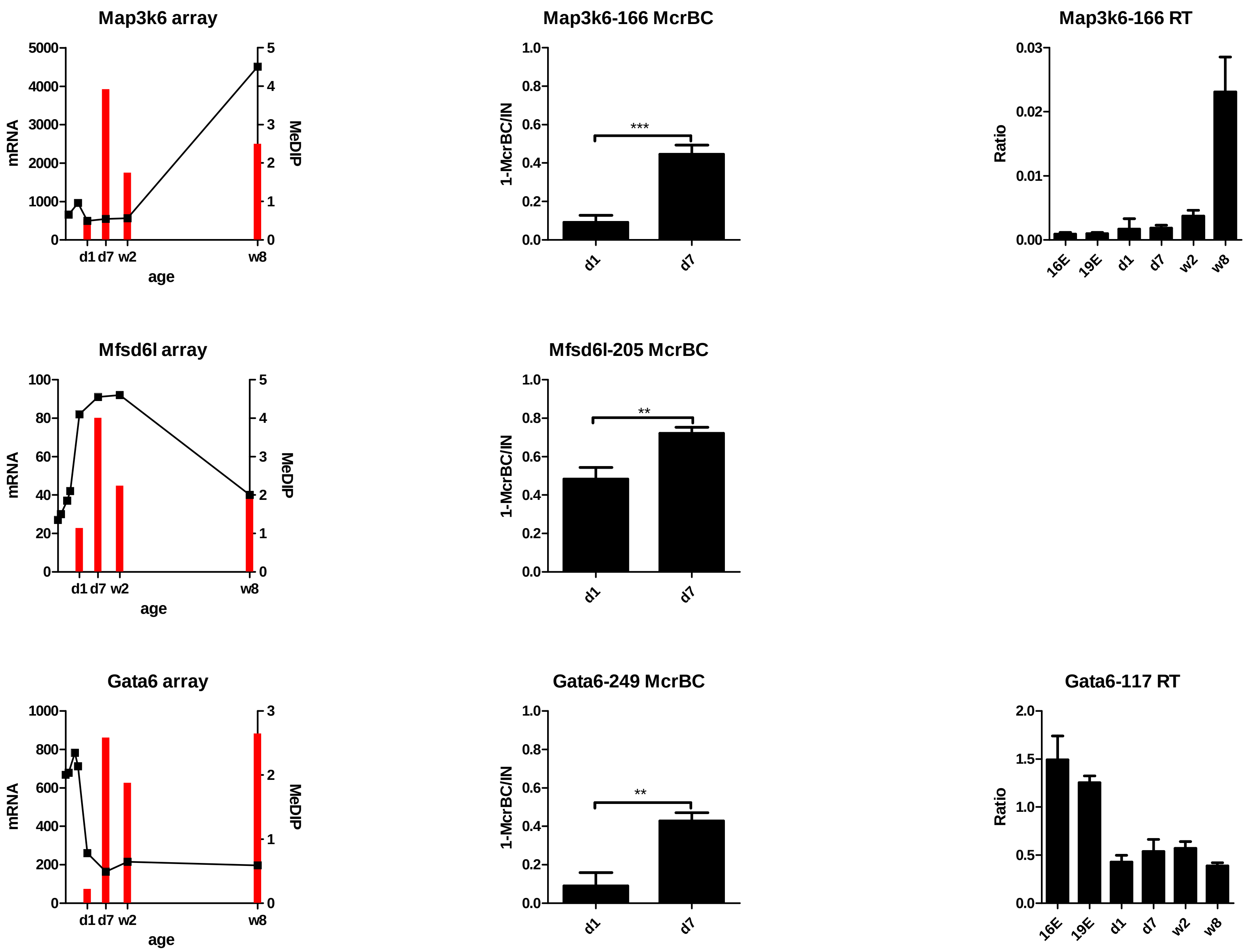
Map3k6 array
mRNA
MeDIP
5000
4000
3000
2000
1000
0
5
4
3
2
1
0
d1 d7 w2
w8
age
Map3k6-166 McrBC
1-McrBC/IN
1.0
0.8
0.6
0.4
0.2
0.0
***
d1
d7
Map3k6-166 RT
Ratio
0.03
0.02
0.01
0.00
16E
19E
d1
d7
w2
w8
Mfsd6l array
mRNA
MeDIP
100
80
60
40
20
0
5
4
3
2
1
0
d1 d7 w2
w8
age
Mfsd6l-205 McrBC
1-McrBC/IN
1.0
0.8
0.6
0.4
0.2
0.0
**
d1
d7
Gata6 array
mRNA
MeDIP
1000
800
600
400
200
0
3
2
1
0
d1 d7 w2
w8
age
Gata6-249 McrBC
1-McrBC/IN
1.0
0.8
0.6
0.4
0.2
0.0
**
d1
d7
Gata6-117 RT
Ratio
2.0
1.5
1.0
0.5
0.0
16E
19E
d1
d7
w2
w8

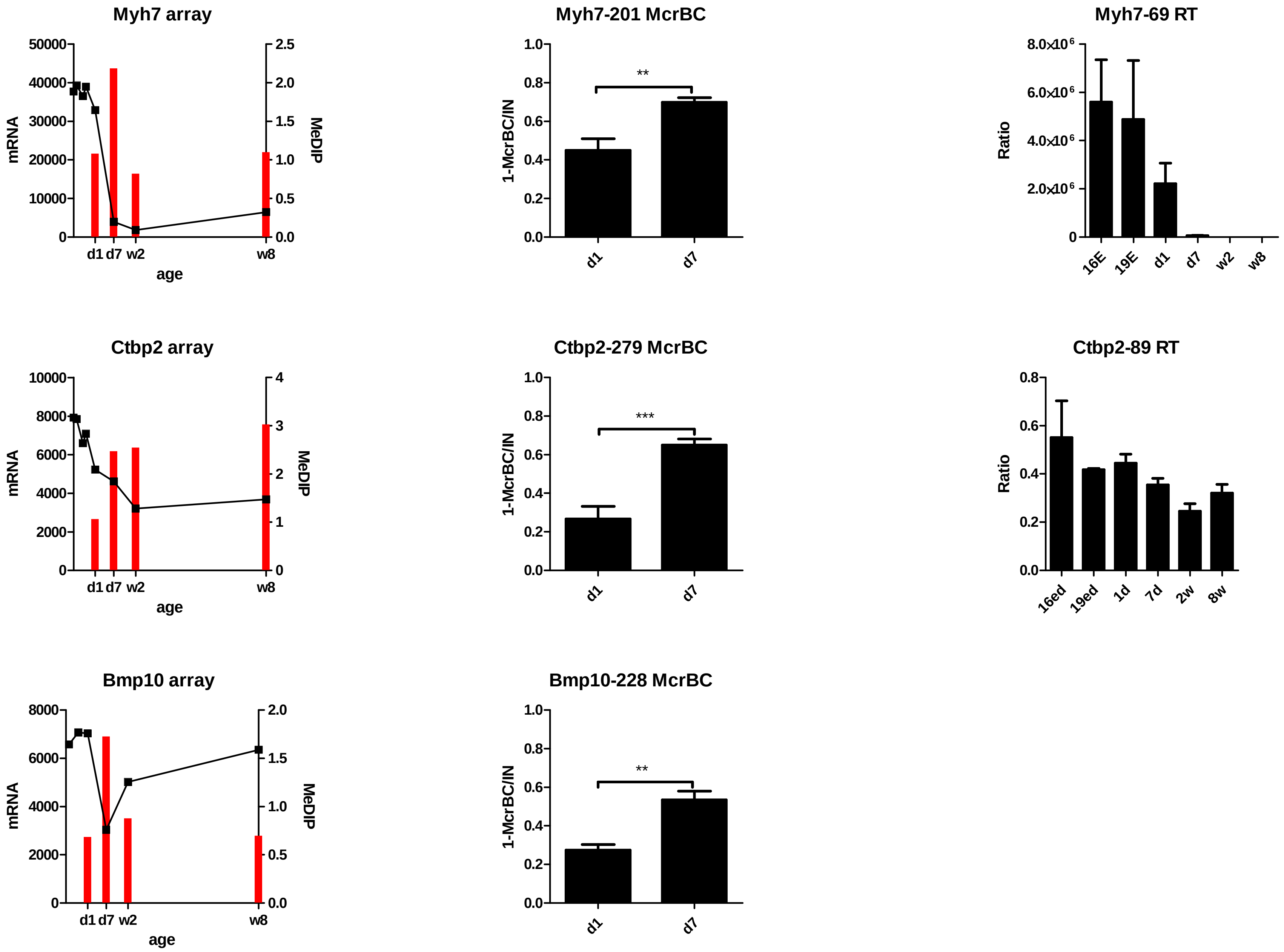
Myh7 array
50000
40000
30000
20000
10000
0
2.5
2.0
1.5
1.0
0.5
0.0
mRNA
MeDIP
d1 d7 w2
w8
age
Myh7-201 McrBC
1.0
0.8
0.6
0.4
0.2
0.0
1-McrBC/IN
**
d1
d7
Myh7-69 RT
8.0x10^6
6.0x10^6
4.0x10^6
2.0x10^6
0
Ratio
16E
19E
d1
d7
w2
w8
Ctbp2 array
10000
8000
6000
4000
2000
0
4
3
2
1
0
mRNA
MeDIP
d1 d7 w2
w8
age
Ctbp2-279 McrBC
1.0
0.8
0.6
0.4
0.2
0.0
1-McrBC/IN
***
d1
d7
Ctbp2-89 RT
0.8
0.6
0.4
0.2
0.0
Ratio
16ed
19ed
1d
7d
2w
8w
Bmp10 array
8000
6000
4000
2000
0
2.0
1.5
1.0
0.5
0.0
mRNA
MeDIP
d1 d7 w2
w8
age
Bmp10-228 McrBC
1.0
0.8
0.6
0.4
0.2
0.0
1-McrBC/IN
**
d1
d7

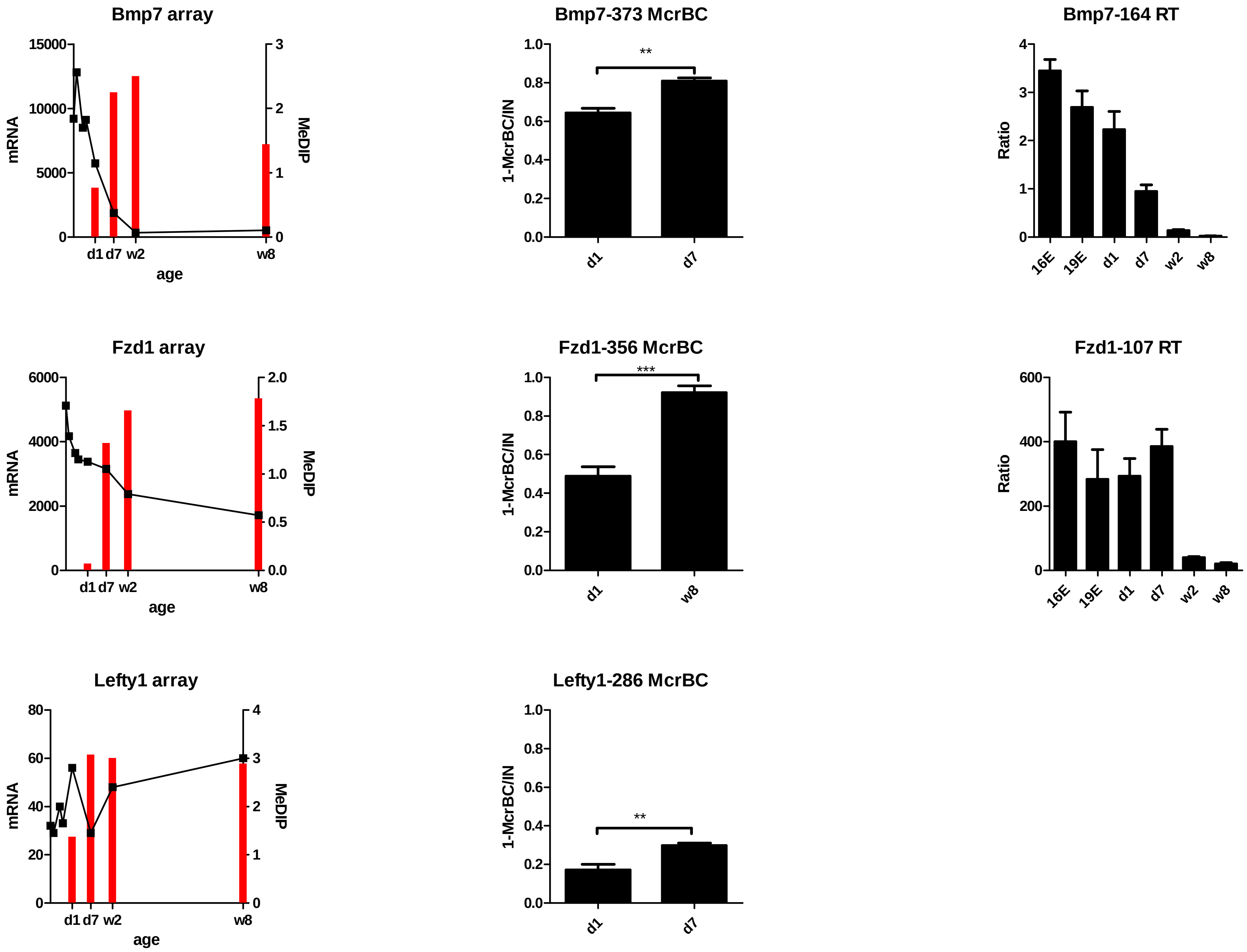

Bmp7 array
15000
10000
5000
0
3
2
1
0
mRNA
MeDIP
d1 d7 w2
w8
age
Bmp7-373 McrBC
1.0
0.8
0.6
0.4
0.2
0.0
1-McrBC/IN
**
d1
d7
Bmp7-164 RT
4
3
2
1
0
Ratio
16E
19E
d1
d7
w2
w8
Fzd1 array
6000
4000
2000
0
2.0
1.5
1.0
0.5
0.0
mRNA
MeDIP
d1 d7 w2
w8
age
Fzd1-356 McrBC
1.0
0.8
0.6
0.4
0.2
0.0
1-McrBC/IN
***
d1
w8
Fzd1-107 RT
600
400
200
0
Ratio
16E
19E
d1
d7
w2
w8
Lefty1 array
80
60
40
20
0
4
3
2
1
0
mRNA
MeDIP
d1 d7 w2
w8
age
Lefty1-286 McrBC
1.0
0.8
0.6
0.4
0.2
0.0
1-McrBC/IN
**
d1
d7

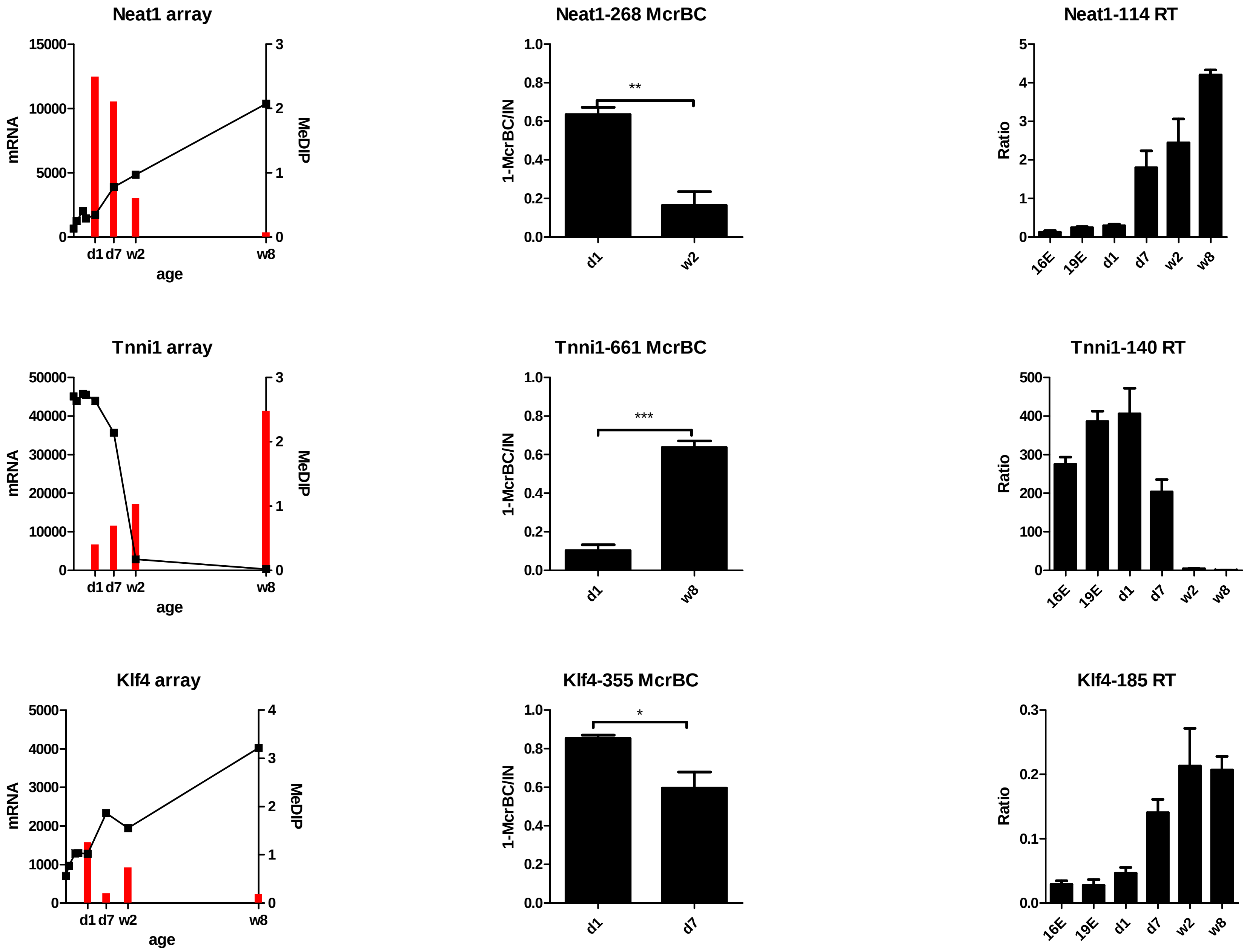
Neat1 array
mRNA
MeDIP
age
d1 d7 w2 w8
Neat1-268 McrBC
1-McrBC/IN
**
d1
w2
Neat1-114 RT
Ratio
16E 19E d1 d7 w2 w8
Tnni1 array
mRNA
MeDIP
age
d1 d7 w2 w8
Tnni1-661 McrBC
1-McrBC/IN
***
d1
w8
Tnni1-140 RT
Ratio
16E 19E d1 d7 w2 w8
Klf4 array
mRNA
MeDIP
age
d1 d7 w2 w8
Klf4-355 McrBC
1-McrBC/IN
*
d1
d7
Klf4-185 RT
Ratio
16E 19E d1 d7 w2 w8

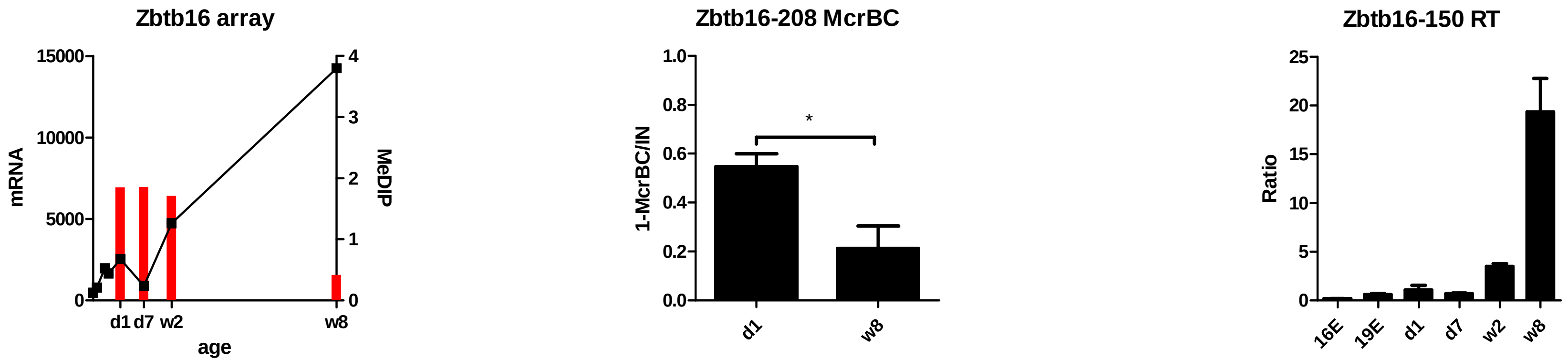

Supplemental file F2. qPCR validation of microarray results – complete results.

The left panels show DNA methylation and gene expression microarray results represented by red bars and black markers, respectively; DNA methylation is expressed as MeDIP enrichment and gene expression levels as normalized microarray signals. The black markers preceding that of neonatal d1 represent gene expression levels in embryonic hearts E16, E18, E19, E20.
The middle panels present DNA methylation levels estimated with Methylation Dependent Restriction Digestion followed by qPCR (MDRE-qPCR) where the methylation levels correspond to the amounts of DNA undigested by CpG methylation dependent McrBC enzyme (1-(McrBC/Input).
The right panels demonstrate transcript levels determined with qPCR as the ratios to the reference transcript of the Tbp gene (TATA binding protein gene).
The microarray results were determined for pooled samples of 3 mice.
The qPCR results represent average values obtained for three individuals for each developmental time-point.
The statistical significance has been determined with two-tailed heteroscedastic Student's t-test.
